# Supplementary material for: MIReVTD, a minimum information standard for reporting vector trait data
Source: Gigascience. 2026 Feb 28;15:giag020. doi: 10.1093/gigascience/giag020 (PMC13122843; doi:10.1093/gigascience/giag020)
Supplement: giag020_GIGA-D-25-00263_Revision_1 [file giag020_giga-d-25-00263_revision_1.pdf]

GigaScience

MIReVTD, a Minimum Information Standard for Reporting Vector Trait Data

--Manuscript Draft--

**Manuscript Number:**	GIGA-D-25-00263R1
**Full Title:**	MIReVTD, a Minimum Information Standard for Reporting Vector Trait Data
**Article Type:**	Review
**Funding Information:**	National Science Foundation (NSF-DBI 2016265) — Professor Sadie Jane Ryan<br>National Science Foundation (NSF-DBI 2016264) — Professor Leah R Johnson<br>National Science Foundation (NSF-DBI 2016282) — Dr Samuel SC Rund
**Abstract:**	Vector-borne diseases pose a persistent and increasing challenge to human, animal, and agricultural systems globally. Mathematical modeling frameworks incorporating vector trait responses are powerful tools for assessing risk and predicting vector-borne disease impacts. Developing these frameworks and the reliability of their predictions hinge on the availability of experimentally derived vector trait data for model parameterization and inference of the biological mechanisms underpinning transmission. Trait experiments have generated data for many known and potential vector species, but the terminology used across studies is inconsistent, and accompanying publications may share data with insufficient detail for reuse or synthesis. The lack of data standardization can lead to information loss and prohibit analytical comprehensiveness. Here, we present MIReVTD, a Minimum Information standard for Reporting Vector Trait Data. Our reporting checklist balances completeness and labor intensity to make these important experimental data easier to find and reuse, without onerous effort for scientists generating the data. To illustrate the standard, we provide an example reproducing results from an Aedes aegypti mosquito study.
**Corresponding Author:**	Sadie Jane Ryan<br>University of Florida<br>Gainesville, FL UNITED STATES
**Corresponding Author Secondary Information:**	
**Corresponding Author's Institution:**	University of Florida
**Corresponding Author's Secondary Institution:**	
**First Author:**	Sadie Jane Ryan
**First Author Secondary Information:**	
**Order of Authors:**	Sadie Jane Ryan<br>Paul Huxley<br>Catherine A Lippi<br>Samraat Pawar<br>Lauren Cator<br>Samuel SC Rund<br>Leah R Johnson
**Order of Authors Secondary Information:**	
**Response to Reviewers:**	Full response included in Personal Cover letter as well - content pasted here from letter. Thanks.<br><br>January 24, 2026

Powered by Editorial Manager® and ProduXion Manager® from Aries Systems Corporation

Dear Dr Fan,
Please find enclosed our revised manuscript "MIReVTD, a Minimum Information Standard for Reporting Vector Trait Data" (GIG-D-25-00263), for publication in GigaScience. We appreciate the minor revisions recommended by the reviewers and editor, and detail below our responses (in italics) to all their comments.

Editorial:

All web links and URLs should be given a reference number and included in the reference list rather than within the text of the manuscript. Please remove the URLs, cite them as reference and adjust the order of the reference accordingly. Except for "Abstract" and "Availability of Source Code and Requirements"

We have made the requested changes

Reviewer #1:

The authors propose MIReVTD, a concise minimum-information checklist for reporting vector trait data, motivated by the lack of consistent terminology and metadata that impedes reuse and synthesis across studies. The scope and intent are clearly stated in the Abstract and Introduction, including the emphasis on FAIR principles and the illustrative Aedes aegypti example and VecTraits implementation. Overall, this is a timely, valuable contribution that complements MIReAD (arthropod abundance) and the vector competence minimum data standard, and it will be highly useful to both experimentalists and modellers.

We thank the reviewer for this comment

Major

- It would be highly beneficial to demonstrate the compatibility and added value of the MIReVTD and the VecTraits database to the existing initiatives aiming to collect and structure similar information. The authors mentioned ETS, MIAPPE, and MIReAD but and explicit mapping of the minimum information field alignment will help to place MIReVTD in context and facilitate adoption of this standard.

We appreciate this comment, and in our introduction, we have added further description of how this differs from prior minimum standards at lines 98-102 to clarify the intent. An explicit multi-standard mapping is outside the scope of the current effort.

- The "Axes of Variation" section is strong, but it could be clearer about what constitutes a stressor or condition. It would help to list common confounders such as humidity, photoperiod, diet or food ration and quality, larval density, and light cycle, and to encourage recording fixed or background conditions in separate fields rather than only gradients. This would help avoid ambiguity between variables that are experimentally varied and those that simply describe the environment.

We thank the reviewer for these excellent points. We realise that we had included our description of "fixed or background conditions", which we refer to as 'additional experimental settings' - that is, those not being manipulated - within the description of Axes of Variation. After debating inclusion of another section of explanation outside the original description, we have instead clarified a little further the intent, and the difference (Lines 130-141)

In Figure 2, the second stressor appears to take a fixed value (0.1). This is somewhat confusing because it is not clear whether this field is meant for another gradient (e.g., temperature in the range of 20 to 40 °C in addition to food ration categories), or whether it lists fixed conditions under which the experiment was performed. If it is the latter, it might be more practical to include additional fields for stressors so that all relevant conditions, such as humidity and photoperiod, can be recorded. It would also help to clarify whether a third or further stressor can be added to the table, and how these would appear.

In this specific case, we apologize that this example may create confusion - the stressor that the reviewer refers to is the first treatment level in a series of levels in the experiment. We have updated the figure legend to point out that we only show the first treatment level.

It might in fact be preferable not to distinguish gradients from fixed conditions at all, and instead to treat them uniformly as conditions, each defined with its corresponding unit and uncertainty. This would simplify the structure and prevent confusion about whether a variable was held constant or systematically varied.

As described above, the purpose here is to provide a flexible structure that allows for additional columns. As the reviewer points out, these are all conditions, and we do not a priori distinguish columns as fixed or varying conditions, but leave these specifications to the user/data curator per experiment.

Powered by Editorial Manager® and ProduXion Manager® from Aries Systems Corporation

	- It would highly improve usability and adoption if the standard also recommended ORCIDs for contributors, DOIs for datasets, and an explicit data license (e.g., CC BY/CC0). If this extension is possible, I recommend that the authors add a short "Data licensing & citation" paragraph to the Results section.<br>We appreciate this comment and have added this paragraph (lines 291-299) and described the suggested citation/attributions for the data sets in the database. In short, we include the study DOI (at publication) with each row of data, and a column to identify who entered/imported that row. If the data are a group of data as part of a metadata study or aggregation of data, we recommend citing that DOI, to reflect the effort of the aggregating authors. These citation metadata are automatically generated at download from the database for data users to incorporate into their work. The overall dataset (VecTraits) hosted at VectorByte, is CC-BY-NC, and has its own DOI as cited in the paper.<br>Minor<br>- Line 248: Fig. 2?<br>We thank the reviewer for highlighting a typo on our part. In the first sentence of the section titled "Example dataset," "Fig.1" should reference "Fig 2." instead.<br>- Please update the citation of bayesTPC.<br>bayesTPC is now published and we have updated the reference.<br>- If possible, please provide a code snippet with the data used (in Zenodo or as Supplementary Material) for Fig. 1.<br>Thank you, the code is mentioned in the figure legend and is available at https://vectorbyteorg.github.io/MireVTD-fig-1/<br>- I believe the followings are also relevant to this study and should be mentioned appropriately:<br>- Adams B, Franz N, König-Ries B, et al. TraitBank: Practical semantics for organism attribute data. Semantic Web. 2015;7(6):577-588. doi:10.3233/SW-150190<br>- Kattge, J., Ogle, K., Bönisch, G., Díaz, S., Lavorel, S., Madin, J., Nadrowski, K., Nöllert, S., Sartor, K. and Wirth, C. (2011), A generic structure for plant trait databases. Methods in Ecology and Evolution, 2: 202-213.<br>Thank you for pointing these out, and we have included them as examples of additional trait database efforts.<br>Reviewer #2: I read with interest the manuscript as I wholeheartedly agree there is a strong need for harmonization on reporting quantitative measurements of vector traits, especially for the subsequent development of mathematical models.<br>The paper is well written, and examples are very helpful, particularly the one shown in Figure 1, advocating for the need for the sharing of individual (possibly raw) observations. I have some very minor comments and suggestions.<br>Thank you for the comments<br>Given the broad readership of the journal, I feel the Introduction would benefit from some definitions of what the authors mean by vector and vector-borne diseases, with some examples (WNV, DENV, … up to you).<br>We thank the reviewer for this suggestion and now provide some examples in the Introduction (lines 57-60)<br>It's not very clear to me how the authors' current proposal aligns with what already proposed in Wu et al. 2022 (ref 21). It seems like some sort of extension? Could you please further elaborate on this?<br>In response to this and reviewer 1's comment, we have provided clarification in lines 98-102 of the Introduction. We do not want to detract from presenting the standard itself with lengthy comparisons and elaborations, but appreciate the opportunity to clarify.<br>Regarding latitude and longitude, I think also the coordinate reference system should be standardized (WGS, no UTM or others).<br>As this is a minimum standard for data entry, we err on the side of ensuring the input datum and resolution is specified, which allows for later users to perform their own spatial standardization and conversion steps. We can encourage a standard system, but this is not the intent of this effort.<br>You might provide some examples of online repositories (line 187). Some (like GitHub) might not be perpetually available, differently from (hopefully) others like Zenodo or the Supplementary Materials accompanying the paper. The latter might be preferrable in my opinion.<br>We are not entirely sure to what this comment is referring, but appreciate the thought that online repositories may not be available forever, and we would argue in fact that in many cases Supplementary Material or Appendices can be even shorter lived as

	journals move between publishers, and in our experience, components can become disconnected in the process, and appendices and supplementary can become irretrievable a decade later. (SJR Personal Experience).<br>Figure 1. Please provide the equation of the TPC.<br>We thank the reviewer for this comment. The code for constructing Figure 1 is hosted at https://vectorbyteorg.github.io/MireVTD-fig-1/<br>Please note that Figure 2 currently does not seem to be cited in the main text (perhaps it should be on line 248?). What does "Dataset: 572" mean?<br>We thank the reviewer for highlighting a typo on our part. In the first sentence of the section titled "Example dataset," "Fig.1" should reference "Fig 2." instead.<br>We have clarified in the legend now that Dataset 572 refers to the VectorByte VecTraits data ID number.<br>As currently VecTraits seem the best (and only?) example of what the authors are proposing, perhaps it should be mentioned in the Abstract as well.<br>We appreciate this comment, but feel that the paper itself underscores the point, and could not see an easy way to incorporate this into the Abstract.<br>Lastly, during revision, one author (PH) developed an additional illustrative schematic of the MIReVTD reporting standard, shown here:<br><br>Optional schematic<br><br>Fig. x. Schematic overview of the MIReVTD minimum information standard.<br>Conceptual schematic illustrating the minimum reporting components of MIReVTD (Minimum Information standard for Reporting Vector Trait Data). Vector trait datasets should minimally report (i) Organism information, including vector taxonomy, life stage, sex, and relevant pathogen identity; (ii) Trait description, specifying the trait measured, how it was measured, the least-aggregated values available, and units; and (iii) Axes of variation, describing experimental or observational dimensions (e.g., temperature, resource level, age) across which the trait was measured. These core components are supported by Metadata, including source citation, provenance, and access information, to enable data discovery and attribution. When reported in this form, vector trait data are findable, interpretable, and reusable for synthesis, database integration, and mechanistic modeling applications.<br>While we are aware this is redundant with current narrative content and adds length to the submission, if its inclusion in the manuscript is welcome, we would like editorial input on best placement.<br>We feel that the reviewer feedback facilitated clarification of the manuscript, and hope you now find it suitable for publication in GigaScience.<br><br>Please do not hesitate to contact me, sjryan@ufl.edu for any further information.<br>On behalf of the authors,<br><br>Sincerely,<br><br>Sadie Ryan, PhD<br>Professor, Medical Geography
**Additional Information:**	
**Question**	**Response**
Are you submitting this manuscript to a special series or article collection?	No
**Experimental design and statistics**<br><br>Full details of the experimental design and statistical methods used should be given in the Methods section, as detailed in our Minimum Standards Reporting Checklist. Information essential to interpreting the data presented should be made available	No

Powered by Editorial Manager® and ProduXion Manager® from Aries Systems Corporation

in the figure legends.<br><br>Have you included all the information requested in your manuscript?	
If not, please give reasons for any omissions below.<br><br>as follow-up to "**Experimental design and statistics**<br><br>Full details of the experimental design and statistical methods used should be given in the Methods section, as detailed in our Minimum Standards Reporting Checklist. Information essential to interpreting the data presented should be made available in the figure legends.<br><br>Have you included all the information requested in your manuscript?<br><br>"	This is not using a minimum reporting standard, rather presenting one.
**Resources**<br><br>A description of all resources used, including antibodies, cell lines, animals and software tools, with enough information to allow them to be uniquely identified, should be included in the Methods section. Authors are strongly encouraged to cite Research Resource Identifiers (RRIDs) for antibodies, model organisms and tools, where possible.<br><br>Have you included the information requested as detailed in our Minimum Standards Reporting Checklist?	No
If not, please give reasons for any omissions below.<br><br>as follow-up to "**Resources**	not relevant

A description of all resources used, including antibodies, cell lines, animals and software tools, with enough information to allow them to be uniquely identified, should be included in the Methods section. Authors are strongly encouraged to cite Research Resource Identifiers (RRIDs) for antibodies, model organisms and tools, where possible.<br><br>Have you included the information requested as detailed in our Minimum Standards Reporting Checklist?<br><br>"	
**Availability of data and materials**<br><br>All datasets and code on which the conclusions of the paper rely must be either included in your submission or deposited in publicly available repositories (where available and ethically appropriate), referencing such data using a unique identifier in the references and in the "Availability of Data and Materials" section of your manuscript.<br><br>Have you have met the above requirement as detailed in our Minimum Standards Reporting Checklist?	Yes
GigaScience has policies and guidelines in place for the use of generative AI-writing tools such as ChatGPT. If you have used such writing tools to assist with writing the manuscript this must be declared and cited in the text. Authors should not list AI-writing tools and other AI-assisted technologies as an author or co-author and should acknowledge that they are fully responsible for text generated or refined by AI-writing tools.<p><br><br>A summary of use (particularly in the introduction or among methods) needs to be included at the end of the paper, and the outputs should also be included as a supplementary file hosted in GigaDB or other open repositories. Please <a href=https://academic.oup.com/gigascienc	No

Powered by Editorial Manager® and ProduXion Manager® from Aries Systems Corporation

e/pages/editorial_policies_and_reporting_standards target="_new" > read our guidelines for more information. <p><br><br>By submitting to GigaScience, you are aware of the journal's AI-writing tools policy, and if you have declared use of such tools below, you have acknowledged this where appropriate in your manuscript and have made a summary of use and outputs available. <p><br><b>AI-assisted writing tools have been used in the preparation of this manuscript?	

Manuscript Click here to access/download;Manuscript;MIReVTD Rev1 GigaSci.docx

MIReVTD, a Minimum Information Standard for Reporting Vector Trait Data

Sadie J. Ryan, Quantitative Disease Ecology and Conservation (QDEC) Lab, Department of Geography and the Emerging Pathogens Institute, University of Florida, Gainesville, FL 32610 United States of America. sjryan@ufl.edu | https://orcid.org/0000-0002-4308-6321

Paul J. Huxley, Department of Infectious Disease Epidemiology, School of Public Health, Imperial College London, United Kingdom. p.huxley@imperial.ac.uk | https://orcid.org/0000-0001-9211-9479

Catherine A. Lippi, Quantitative Disease Ecology and Conservation (QDEC) Lab, Department of Geography and the Emerging Pathogens Institute, University of Florida, Gainesville, FL 32610 United States of America. clippi@ufl.edu | https://orcid.org/0000-0002-7988-0324

Samraat Pawar, Department of Life Sciences, Silwood Park, Imperial College London United Kingdom. s.pawar@imperial.ac.uk | https://orcid.org/0000-0001-8375-5684

Lauren Cator, Department of Life Sciences, Silwood Park, Imperial College London, United Kingdom. l.cator@imperial.ac.uk | https://orcid.org/0000-0002-6627-1490

Samuel S.C. Rund (Center for Research Computing, Department of Biological Sciences, and Eck Institute for Global Health, University of Notre Dame, Notre Dame, IN, 46616, United States of America. srund@nd.edu) | https://orcid.org/0000-0002-1701-7787

Leah R. Johnson Department of Statistics, Virginia Tech, 250 Drillfield Drive, Blacksburg, VA 24061, United States of America. lrjohn@vt.edu | https://orcid.org/0000-0002-9922-579X

Abstract

Vector-borne diseases pose a persistent and increasing challenge to human, animal, and agricultural systems globally. Mathematical modeling frameworks incorporating vector trait responses are powerful tools for assessing risk and predicting vector-borne disease impacts. Developing these frameworks and the reliability of their predictions hinge on the availability of experimentally derived vector trait data for model parameterization and inference of the biological mechanisms underpinning transmission. Trait experiments have generated data for many known and potential vector species, but the terminology used across studies is inconsistent, and accompanying publications may share data with insufficient detail for reuse or synthesis. The lack of data standardization can lead to information loss and prohibit analytical comprehensiveness. Here, we present MIReVTD, a Minimum Information standard for Reporting Vector Trait Data. Our reporting checklist balances completeness and labor intensity to make these important experimental data easier to find and reuse, without onerous effort for scientists generating the data. To illustrate the standard, we provide an example reproducing results from an *Aedes aegypti* mosquito study.

Keywords: traits, minimum information, vectors, data, ecoinformatics

Introduction

Biological data are increasing in size and scope, and the means of reporting experimental or measured data are wide-ranging in format - from journals [1], to collections (*e.g.* NEON Biorepository [2]), to sequence repositories (*e.g*. GenBank [3]). The practice of synthesizing data across multiple studies (*e.g.,* exploring patterns such as taxonomic structuring, geographic trends, biotic and abiotic drivers, and trends) relies on a consistency of data reporting, in terms of measurement units, specific IDs, nomenclatures, and well-specified terminology. For example, the use of trait data is widespread in ecological research, underpinning much of the foundational exploration and approaches in ecological and evolutionary mechanisms. Thus, initiatives to standardize the wide variety of available ecological trait data exist [4–6].

The ability to synthesize and reuse data is particularly important for vector-borne disease (VBD) research. The risk of VBDs in people (e.g. mosquitoes transmitting West Nile virus), livestock/animals (e.g. ticks transmitting Lyme disease to a horse), and crops/plants (e.g. aphids transmitting Potato virus Y to potatoes) is currently increasing, in particular due to interactions with climate and land cover change [7–10]. Understanding the shape and pattern of that risk, and potential additional risk for VBDs requires data on the underlying biological mechanisms of transmission. However, amassing the appropriate data to synthesize and analyze these essential model-building blocks can be stymied by the sheer range of terminology, reporting styles, outputs, and a lack of a coherent framework to store them. While multiple databases for vector ecology data of many kinds exist, their scopes vary, as do their accessibility and thus capacity for reuse and synthesis [11]. Traits of arthropod vectors – measurable biological aspects of life-history, behavior, and vector competence – are integral to disentangling the complex mechanisms that underlie VBD transmission [12]. Linking vector traits to transmission dynamics, in turn, is a crucial step in constructing useful mathematical frameworks and mechanistic models to predict disease dynamics and risk [13–15]. While mechanistic models are undeniably powerful tools for estimating

disease risk, the challenges of building and parameterising such models are also widely acknowledged. Chief among these is the sheer amount of data needed to parameterize models in biologically meaningful ways. The empirical data needed to derive realistic parameter estimates are typically collected through extensive experimentation in controlled laboratory settings. Thus, obtaining useful vector trait data, such as measurements of vector competence, fecundity, longevity, etc. across abiotic gradients (*e.g.*, temperatures), is both financially and logistically costly to obtain. There is a clear benefit to leveraging large datasets synthesizing information from many sources (e.g.[14,16–18]), yet the lack of a minimum information standard for reporting data generated by vector trait experiments impedes our capacity for aggregating data across collection efforts.

To ensure usability by the broader scientific community, datasets should adhere to FAIR Principles – Findable, Accessible, Interoperable, and Reusable – which are key components of good data management practices [19]. Generally, the information shared will comprise two components, i) data, or measured traits and outcomes generated by experiments, and ii) metadata, or information about the origin of the data.

Here we present MIReVTD (Minimum Information standard for Reporting Vector Trait Data), a minimum information standard developed to accommodate vector trait experimental data within a flexible, transparent, and well-documented database backbone, with accompanying metadata to facilitate data sharing and usability. Minimum information standards define a checklist of information minimally required to understand and reuse a biological dataset. They do not prescriptively define a specific set of field names or data types [20], but it is useful to provide examples in practice (data standards) which do, as illustration. Examples of minimum information standards include MIAPPE (Minimum Information About a Plant Phenotyping Experiment) [21]; MIReAD (Minimum Information standard for Reporting arthropod Abundance Data) [22]; and Wu

et al.'s minimum data standard for vector competence experiments [23]. The current effort differs from the previous minimum information standards, as it is designed to facilitate data sharing and reuse of vector trait experimental evidence rather than infection experiments or abundance records. The minimum information standard we report here arose from efforts comprising two long-term research projects, one of which sought to define "what is a trait?" for disease vectors [12], and the other is part of a long-term informatics project, VectorByte [24]), that is building a database (VecTraits [25],) containing the answer. The VecTraits database and format is an exemplar implementation and operationalization of the minimal information standard presented here, accommodating the minimum information needed, while providing flexibility for expanding fields to iterate across multiple axes of variation [25].

Results

Among vector trait experiments and observations, there is considerable variation in vector trait data generated by independent studies, including which traits are measured, and the conditions under which they are measured. Due to the inherent complexity of data generated by vector trait experiments, this is not intended as a template for data collection, but rather a guide to what minimum information must be included when reporting outcomes, to ensure secondary use of data. At the most basic level, the minimum descriptor set for vector traits to maximize usability across studies are as follows:

Organism: The genus and species of vector being studied and, if known, particular subspecies or lab strain. For individually measured data, this may also include some unique identifier to designate each individual or replicate (for example when multiple traits or timepoints are measured on the same individual organism). In transmission experiments, species or strain of pathogen must also be reported. Sex and life stage/age of the organism should be included.

Trait Description: The vector trait being studied, how it was measured, the units of measurement used, and the frequency of observations. Ideally data should be in the least aggregated form available (*e.g.*, measurements on individuals instead of means across individuals). When only means (or other summaries) are available, metrics of variability (*e.g.*, standard error) and their descriptions (including sample sizes) should be included.

Axes of Variation: Refers to the experimental or observational dimensions along which a focal trait is intentionally measured or contrasted, rather than incidental conditions or derived traits. Specify which abiotic (*e.g.*, temperature) or biotic (*e.g.*, food source) gradients were incorporated into the study, the frequency at which observations were made, and the units by which this variation was recorded. Multiple such covariates, many of which are biological "stressors" may be incorporated into each study. The sampling design should also be specified. For example, were trait measurements taken on multiple individuals at a single point in time, or were individuals tracked through time and measured across a gradient (several distinct treatments) such as time or temperature? Note that well annotated granular data make experimental design self-evident. Any additional experimental conditions (*e.g.*, ambient temperature when temperature is not manipulated) should also be recorded. This will be included in an additional data column, leveraging the flexibility of this design.

The 3-component minimum information we have outlined here is expanded upon in Table 1 and in Box 1. Table 1 gives examples of data fields and the types of details recommended, while Box 1 provides some more general suggestions and guidelines on formatting.

Table 1. Example data fields to capture minimum descriptors for vector trait experiments.

Descriptor	Field(s)	Details	Recommendations	Examples
**Organism**	Vector taxonomy	Genus and species of vector being studied, and if available, subspecies or laboratory strain	Be as specific as possible<br><br>Do not use abbreviations<br><br>If known, include lab or colony strain name<br><br>If relevant, a lab strain identification name / number / barcode	“*Aedes aegypti*”<br><br>“*Culex quinquefasciatus* Sebring colony”<br><br>“*Delphacodes kuscheli*”<br><br>“NR-44077 *Rhodnius prolixus*, Strain CDC”
	Unique identifier	Designation of individuals or replicates in experiments, if applicable	Internal naming convention for study<br><br>Can be used to link related observations from a series or lab experiment<br><br>For example, a trait may be measured, on the same animal, at multiple ages - linked together by a unique (animal) identifier	“Mosquito1”<br><br>“tick 45”

	Pathogen taxonomy<br><br>(when relevant, *e.g.* transmission studies)	Genus, species, and strain (if known) of pathogen	Be as specific as possible<br><br>Do not use abbreviations<br><br>If known, include viral or pathogen strain<br><br>If relevant, a lab strain identification name / number	"*Plasmodium falciparum* (N54 strain)"<br><br>"West Nile virus"<br><br>"Dengue virus DENV-4 (strain H241)"<br><br>"MRA-578 *Plasmodium falciparum*, Strain D10 PfM3' [D10-PfM3' (wt MSP-1 replacement)]"
	Age / life stage	The age / life stage that was assayed	Be as specific as possible<br><br>Do not use abbreviations	"L1 larvae"<br><br>"3-4 day old post eclosion adults"<br><br>"nymphs"
	Sex	The sex of the organism that was assayed		"males"<br><br>"unknown"<br><br>"mixed"

**Trait Descriptio n**	Vector trait	A detailed description of the trait being studied	Be as specific as possible, if appropriate specify life stage to avoid confusion<br><br>Avoid abbreviations	“mortality”<br><br>“lifespan”<br><br>“Fecundity”<br><br>“development time - hatch to adult”
	Value	A numerical measurement of the trait being studied	Provide units in separate field, or in column heading	45<br><br>17<br><br>0.25
	Units of measureme nt	The units of measurement used to record values for trait data	For percentages or proportions, the number of “successes” (numerator) and total observations (denominator) should be recorded.<br><br>For rates, length of time intervals over which the trait is measured should be reported.	“days”<br><br>“eggs laid”<br><br>“percent mortality”<br><br>“LT50”

	Study location	Geographical location where field study was conducted, or where samples were collected	This should be recorded only for experiments where location could influence results, for example field studies or source location of collected individuals<br><br>e.g., locations where lab tests were run are not part of the experimental design, and may inadvertently suggest that local specimen strains were used<br><br>Be as detailed as possible, reporting latitude and longitude if available	
**Axes of Variation**	Example field(s):	The abiotic experimental factor (variables) at which trait observations are measured	Common gradients include:<br><br>Temperature<br>Relative humidity<br>Photoperiod<br><br>Location<br>Date	Separate different axes of variation into different columns
	Units of measurement	The units of measurement for experimental factor		“Degrees celsius”<br><br>“Age, days post emergence”

Box 1. General suggestions and guidelines for reporting traits data

1. Do not use abbreviations in data fields and especially not in field names - they introduce uncertainty.
2. Avoid the use of more than one natural reporting language (e.g., mixing English and Standard Mandarin) as this can result in interpretation errors.
3. Use numeric dates, preferably ISO 8601 format (*e.g.* YYYY-MM-DD)
4. Provide data that are machine readable. For example, numerical data should be separated from its units into different fields. For more information on suggestions for reporting units, see Hanisch et al.[26]
5. Avoid diacritics (accent marks) and other special characters as they often lead to problems in reuse as some systems will not handle them correctly due to encoding differences.
6. When providing the geographic location of data collection (for example when reporting a trait value across a geographic range), be as detailed as possible. Latitude and longitude are preferred, if available. *NOTE: Do not inadvertently indicate that local specimen strains were used by providing the location of a laboratory, when samples used in the experiment were collected elsewhere.*
7. The less processed the data are, the more reusable the data will be. We advocate providing per-organism data when possible. For example, report data on the lifespan of each animal, instead of "average lifespan" or report each measure of a multiple-measure experimental design, linked with an appropriate unique (*e.g.* animal) identifier (e.g., report number of eggs laid each day by each individual mosquito).
8. Save files in a text-based, non-proprietary format, preferably as a .csv file.

In Summary, if a dataset is well reported and formatted, with all the minimal information, a secondary user should be able to understand from the data what experiment was performed just

from looking at the data, including what organism was assayed, what was measured, under what condition(s), and what was the axis of variation.

The above outlines the bare minimum information needed to ensure that datasets are coherent and reusable beyond the original study. However, vector trait data can quickly become complex, necessitating additional detail and clarification to maintain their utility. In these instances, specificity matters. For example, when measuring wing length, along which axis is the measurement taken? When recording body mass, is it dry or wet mass, is weight taken for an intact insect, or is it wingless/legless mass, and for a single individual or averaged across multiple individuals? In addition to the basic required data fields, **additional Trait Description fields** should be added as needed to capture the dimensionality of information generated in a study. Further, there may be additional traits recorded in a study, which may influence the first trait described. In each of these instances, we can iterate the basic data inputs of Trait Description and Axis of Variation.

Study and project metadata are equally vital for maintaining usability and interoperability of primary vector trait data. At minimum, reported metadata should include a full citation of the data source (*e.g.*, this is typically a published paper), the name and contact for the person uploading the dataset to an online repository, and if relevant, the date on which any embargo on the dataset is lifted (*e.g.* for publication purposes). We also advocate reporting data in the least-processed, most raw form. Generally, this means that for every experimental measurement or observation, that data point is represented individually in the reported data - and not as averages or derived values. As examples: reporting the lifespan of each individual animal in a mortality experiment instead of an LT50 or mean longevity.

The importance of disaggregated data: an illustration

As an illustration of the importance of reporting individual level rather than group level information from vector trait experiments for mechanistic model parameterization, Figure 1 shows the difference in estimates generated for predicted juvenile development rate as a function of temperature, using group averages versus individual level data from Huxley et al.'s [18] study on *Aedes aegypti*. Both datasets here are fitted with the same parametric function for the thermal performance curve (TPC), using the same Bayesian fitting algorithm and the same low information priors. Note how both the peak rate and the temperature bound estimates are impacted when averages are used in place of the original individual data, and the difference in errors around those estimates. In this case, strongly informative priors for the TPC model parameters (i.e., T_{min}, T_{max}) would need to be set when fitting to the averages in order to obtain fits that are comparable to the fit obtained for the individual level data. That is, extra outside information would need to be included to compensate for the loss of information that occurred when the averages were taken. This example illustrates a general principle underlying MIReVTD: aggregation is irreversible loss of information for mechanistic inference.

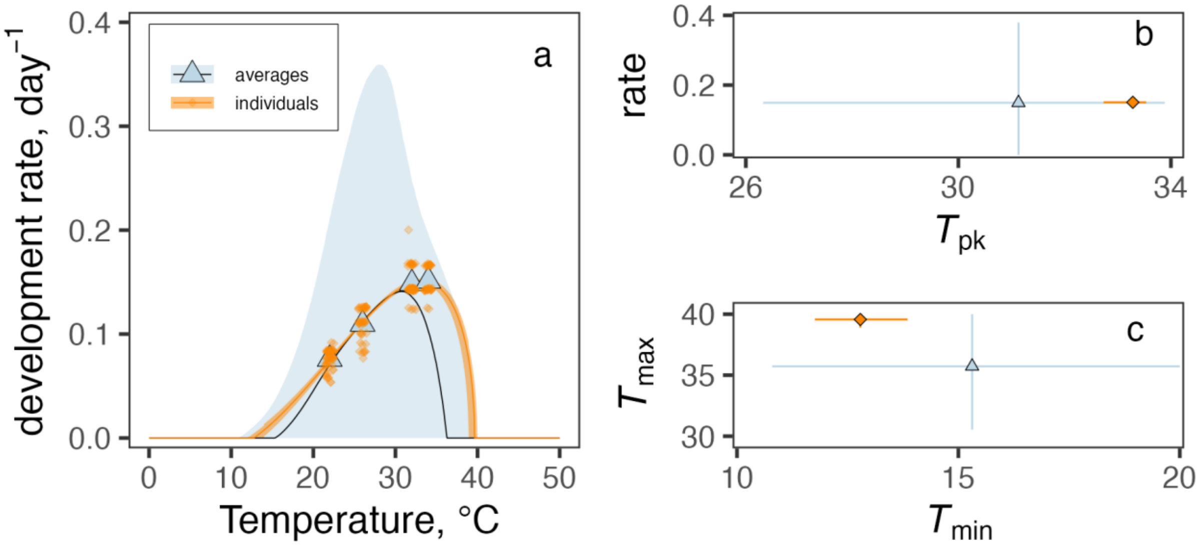

Fig. 1. a. Example of some differences that can arise when TPCs (thermal performance curves) are fitted to single-point averages versus individual-level observations when priors are set to be weakly informative (both fits use the same priors for all parameters). The trait here is juvenile development rate (inverse of duration from hatching to adult eclosion). Blue triangles denote averages; the blue bounds are the 95% credible intervals for the fitted central response (median). Orange diamonds are individual observations (slightly jittered); the orange bounds are the 95% credible intervals for the fitted central response (median). b. Differences between predicted development rate at its T_{pk} (i.e., the temperature at which a trait reaches its highest value) for TPCs fitted to averages (triangles) and individual observations (diamonds). c. Differences between predicted T_{min} and T_{max} for TPCs fitted to averages (triangles) and individual observations (diamonds). Points in b and c are median posterior estimates. Error bars in b. and c. are 95% highest posterior density (HPD) intervals for each parameter. TPCs were fitted to data from Huxley et al. 2022 [18] using the bayesTPC package in R [27]. Code to reproduce this figure is available at https://vectorbyteorg.github.io/MireVTD-fig-1/

VecTraits Database

The VectorByte initiative [24] has worked to establish a global and openly accessible data hub to support vector research, which itself was inspired by earlier work on thermal performance traits from the BioTraits project [28]. An outcome of VectorByte was the release of the VecTraits Database [25], an online platform for open hosting and sharing of biological vector trait data. Here, we use VecTraits to demonstrate an implementation of the minimum information standard, the corresponding data standard, and additional suggested metadata capture. For data input, certain fields are required by VecTraits, by design, in part to maintain interoperability with other databases (especially BioTraits [29]). Fields are also required to satisfy the need for minimum information for reuse. Beyond the first set of required entry fields, iterations of fields for minimum standards (*e.g.*, OrganismID, Trait Description) can be entered into VecTraits as needed, labeled as

‘interactors’. For example, vector competence and transmission studies should also include species or strain of pathogen used in the study, and this information would be recorded in VecTraits through the “interactor2” fields where appropriate. The interactor2 field is not required for uploading datasets into VecTraits because this may not apply to all experiments, but for studies that include pathogens, the minimum information standard described here indicates that this information is required to be reported. VecTraits thus tries to strike a balance between requiring sufficient fields be present and correctly input, and the flexibility to expand necessary columns of input to accommodate multiple axes of variations that may be included in a study or set of experiments. The current list of VecTraits field names and column definitions is spelled out, including examples of the data one would enter, the data format (TEXT, INTEGER, BOOLEAN, *etc.*), and restrictions on format (e.g. Not null, length <= 255 characters), can be found at [30]. At upload, VecTraits has a series of checks and will display error flags for the user, facilitating minimal required formatting.

Example Dataset

Here we present an exemplar dataset retrieved from the VecTraits database to illustrate how this minimum information standard may be applied in practice (Fig 2). These data originated from a study by Huxley et al. [31] on the effects of larval competition and resource depletion on the temperature dependence of maximal population growth rates in the *Aedes aegypti* mosquito. This example demonstrates how the VecTraits database, with its own naming conventions and data entry fields, still complies with the minimum information standard while maintaining the flexibility needed to host data generated through complex experimental designs. By avoiding the use of a fixed template for all data columns, there is enough adaptability in the data entry process to expand columns as needed to capture requisite aspects of a given study that do not unilaterally apply to every experiment on vector traits. In this example, lifespan was recorded across a temperature gradient, which is a reportable “Axis of Variation” under the minimum information

standard. This study also recorded juvenile lifespan (the duration from hatch to death or adult eclosion for all individuals in each sample population) at four initial resource concentration levels, which represents an additional "Axis of Variation" that is reportable under the standard, but is not universally applicable to most studies. In addition to meeting minimal information/data requirements, this example also highlights the collection of adequate metadata; here, information on the published study where the data originated is provided, as well as the name of the user who submitted the dataset to the VecTraits database. Note that while other data were collected in this study, including development time, longevity, and survival, these were entered as unique datasets with bespoke columns to reflect the dimensionality of the traits being measured, though these datasets are still linked through common metadata fields (*i.e.*, citation and DOI).

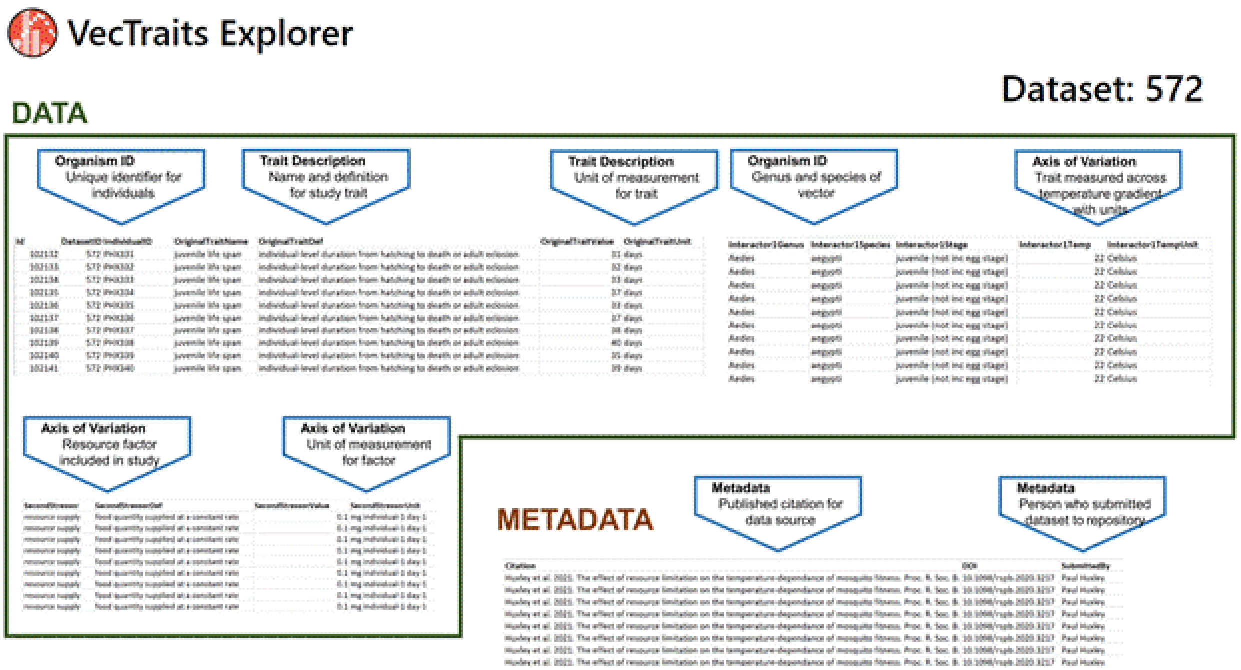

Fig. 2. Example of data in VecTraits for a study measuring juvenile lifespan of *Aedes aegypti*. The trait in this dataset is juvenile life span and it is specified that this refers to surviving until death or adult eclosion. Each row contains a trait measure, number of days alive, for each individual mosquito in the experiment. This study included two stressors (temperature and

resource level - here we show the first level (0.1)) and these are indicated as axes of variation. Measures and units are specified separately. The full citation is included to support source attribution. Example data from Huxley et al [31] as stored in VecTraits dataset number 572 [32].

Data licensing & citation

In brief, in VecTraits [25], we include the study DOI (at publication) with each row of data, and a column to identify who entered/imported that row of data. If the data are grouped as part of a metadata study or aggregation of data, we recommend citing that DOI as well, to reflect the effort of the aggregating authors. These citation metadata are automatically generated at download from the database for data users to incorporate into their work. A pop-up window recommends using the citations provided and also contains the licensing information. The overall dataset (VecTraits) hosted at VectorByte as VecTraits Explorer, is CC-BY-NC, and has its own DOI [25].

Discussion

Establishing minimum information standards for reporting and sharing vector traits data is necessary for maintaining the 'reusability' and FAIR-ness of experimental data. By emphasizing which elements of a study must be reported to ensure that datasets are usable beyond the original study, as opposed to providing a set template for data collection, the minimal information standard we present here has the necessary flexibility to work with the multitude of experimental designs used to capture trait data, which are incredibly varied in purpose and format. Standardizing the reportable components of shared datasets will benefit the broader community of vector-borne disease researchers, particularly those whose work relies on experimentally derived data to parameterize models. The incorporation of variable trait data into modeling frameworks can deepen our understanding of transmission dynamics and expand the capacity for accurate disease modeling. Yet data-hungry methodological efforts are too often hindered by a lack of

empirical data, which can result in unrealistic model predictions. Laboratory experiments designed to accurately measure vector traits across various Axes of Variation can be logistically demanding and resource intensive, effectively capping the sample size that is obtained from any single experiment. The need for empirical data to support VBD research has not gone unnoticed, and in recent years there have been great advances in the collection of large vector traits datasets, owing to government initiatives, innovations in empirical data collection, and the development of open data repositories. The increasing capacity to collect empirical data, and pool those observations across studies, underscores the pressing need for a cohesive set of minimum information data standards to facilitate secondary data analysis and promote FAIR Principles[19] in data sharing. MIReVTD and platforms such as VecTraits are an important step towards meeting this need. We note that MIReVTD does not enforce ontologies at this stage, is not designed to resolve taxonomic disputes, and cannot guarantee data quality, only interpretability. Further, some legacy datasets cannot be fully compliant with the intents of MIReVTD standard. As the data continue to grow in quantity, and our capacity to leverage those data to explore scientific questions, refine assumptions and generate new knowledge, the importance of cohesion, consistency and maintaining FAIR principles will also grow.

Funding

Several authors were supported by CIBR: VectorByte: A Global Informatics Platform for studying the Ecology of Vector-Borne Diseases (SJR and CAL by NSF-DBI 2016265, LRJ and PH by NSF-DBI 2016264 and SSCR by NSF-DBI 2016282).

Authors' contributions

All authors were responsible for conceiving the paper; SJR, PH, and CAL wrote the initial draft; PH performed the disaggregated data example. All authors contributed to and edited the final version.

Competing Interests

The authors declare no competing interests

References

1. Shimabukuro P, Groom Q, Fouque F, Campbell L, Chareonviriyaphap T, Etang J, et al.. Bridging Biodiversity and Health: The Global Biodiversity Information Facility's initiative on open data on vectors of human diseases. *GigaByte*. 2024; doi: 10.46471/gigabyte.117.

2. Thibault KM, Laney CM, Yule KM, Franz NM, Mabee PM. The US National Ecological Observatory Network and the Global Biodiversity Framework: national research infrastructure with a global reach. *J Ecol Environ*. The Ecological Society of Korea; 2023; doi: 10.5141/jee.23.076.

3. Sayers EW, Bolton EE, Brister JR, Canese K, Chan J, Comeau DC, et al.. Database resources of the national center for biotechnology information. *Nucleic Acids Res*. Oxford University Press (OUP); 2022; doi: 10.1093/nar/gkab1112.

4. Schneider FD, Fichtmueller D, Gossner MM, Güntsch A, Jochum M, König-Ries B, et al.. Towards an ecological trait-data standard. *Methods Ecol Evol*. Wiley; 2019; doi: 10.1111/2041-210x.13288.

5. Parr CS, Schulz KS, Hammock J, Wilson N, Leary P, Rice J, et al.. TraitBank: Practical semantics for organism attribute data. *Semantic Web*. SAGE PublicationsSage UK: London, England; 2015; doi: 10.3233/SW-150190.

6. Kattge J, Ogle K, Bönisch G, Díaz S, Lavorel S, Madin J, et al.. A generic structure for plant trait databases. *Methods in Ecology and Evolution*. John Wiley & Sons, Ltd; 2011; doi: 10.1111/j.2041-210X.2010.00067.x.

7. MacDonald AJ, Mordecai EA. Amazon deforestation drives malaria transmission, and malaria burden reduces forest clearing: a retrospective study. *The Lancet Planetary Health*. thelancet.com; 2019;

8. O'Neill L, Gubbins S, Reynolds C, Limon G, Giorgakoudi K. The socioeconomic impacts of Rift Valley fever: A rapid review. *PLoS Negl Trop Dis*. Public Library of Science (PLoS); 2024; doi: 10.1371/journal.pntd.0012347.

9. Combs MA, Kache PA, VanAcker MC, Gregory N, Plimpton LD, Tufts DM, et al.. Socio-ecological drivers of multiple zoonotic hazards in highly urbanized cities. *Glob Chang Biol*. Wiley; 2022; doi: 10.1111/gcb.16033.

10. Fagre AC, Cohen LE, Eskew EA, Farrell M, Glennon E, Joseph MB, et al.. Assessing the risk of human-to-wildlife pathogen transmission for conservation and public health. *Ecol Lett*. 2022; doi: 10.1111/ele.14003.

11. Lippi CA, Rund SSC, Ryan SJ. Characterizing the Vector Data Ecosystem. *J Med Entomol*. Oxford Academic; 2023; doi: 10.1093/jme/tjad009.

12. Cator LJ, Johnson LR, Mordecai EA, Moustaid FE, Smallwood TRC, LaDeau SL, et al.. The Role of Vector Trait Variation in Vector-Borne Disease Dynamics. *Front Ecol Evol*. 2020; doi: 10.3389/fevo.2020.00189.

13. Mordecai EA, Caldwell JM, Grossman MK, Lippi CA, Johnson LR, Neira M, et al.. Thermal biology of mosquito-borne disease. *Ecology Letters*. Wiley; 2019; doi: 10.1111/ele.13335.

14. Shocket MS, Verwillow AB, Numazu MG, Slamani H, Cohen JM, El Moustaid F, et al.. Transmission of West Nile and five other temperate mosquito-borne viruses peaks at temperatures between 23°C and 26°C. *Elife*. 2020; doi: 10.7554/eLife.58511.

15. Lim A-Y, Jafari Y, Caldwell JM, Clapham HE, Gaythorpe KAM, Hussain-Alkhateeb L, et al.. A systematic review of the data, methods and environmental covariates used to map Aedes-borne arbovirus transmission risk. *BMC Infect Dis*. Cold Spring Harbor Laboratory Press; 2023; doi: 10.1186/s12879-023-08717-8.

16. Brass DP, Cobbold CA, Purse BV, Ewing DA, Callaghan A, White SM. Role of vector phenotypic plasticity in disease transmission as illustrated by the spread of dengue virus by Aedes albopictus. *Nat Commun*. Springer Science and Business Media LLC; 2024; doi:

10.1038/s41467-024-52144-5.

17. Pawar S, Huxley PJ, Smallwood TRC, Nesbit ML, Chan AHH, Shocket MS, et al.. Variation in temperature of peak trait performance constrains adaptation of arthropod populations to climatic warming. *Nat Ecol Evol*. 2024; doi: 10.1038/s41559-023-02301-8.

18. Huxley PJ, Murray KA, Pawar S, Cator LJ. Competition and resource depletion shape the thermal response of population fitness in Aedes aegypti. *Commun Biol*. Springer Science and Business Media LLC; 2022; doi: 10.1038/s42003-022-03030-7.

19. Wilkinson MD, Dumontier M, Aalbersberg IJJ, Appleton G, Axton M, Baak A, et al.. The FAIR Guiding Principles for scientific data management and stewardship. *Sci Data*. 2016; doi: 10.1038/sdata.2016.18.

20. Taylor CF, Field D, Sansone S-A, Aerts J, Apweiler R, Ashburner M, et al.. Promoting coherent minimum reporting guidelines for biological and biomedical investigations: the MIBBI project. *Nat Biotechnol*. Springer Science and Business Media LLC; 2008; doi: 10.1038/nbt.1411.

21. Krajewski P, Chen D, Ćwiek H, van Dijk ADJ, Fiorani F, Kersey P, et al.. Towards recommendations for metadata and data handling in plant phenotyping. *J Exp Bot*. Oxford University Press (OUP); 2015; doi: 10.1093/jxb/erv271.

22. Rund SSC, Braak K, Cator L, Copas K, Emrich SJ, Giraldo-Calderón GI, et al.. MIReAD, a minimum information standard for reporting arthropod abundance data. *Sci Data*. 2019; doi: 10.1038/s41597-019-0042-5.

23. Wu VY, Chen B, Christofferson R, Ebel G, Fagre AC, Gallichotte EN, et al.. A minimum data standard for vector competence experiments. *Sci Data*. Nature Publishing Group; 2022; doi: 10.1038/s41597-022-01741-4.

24. VectorByte (VecTraits and VecDyn databases). VectorByte. http://www.vectorbyte.org Accessed 2025 Dec 3.

25. Johnson LR, Cator L, Rund SSC, Ryan S, Huxley PJ, Pawar S: VecTraits Explorer.

University of Notre Dame; https://doi.org/10.7274/28020782 (2023).
26. Hanisch R, Chalk S, Coulon R, Cox S, Emmerson S, Flamenco Sandoval FJ, et al.. Stop squandering data: make units of measurement machine-readable. *Nature*. Springer Science and Business Media LLC; 2022; doi: 10.1038/d41586-022-01233-w.
27. Sorek S, Smith JW Jr, Huxley PJ, Johnson LR. bayesTPC: Bayesian inference for Thermal Performance Curves in R. *Methods in Ecology and Evolution*. Cold Spring Harbor Laboratory; 2025; doi: 10.1111/2041-210X.70004.
28. Dell AI, Pawar S, Savage VM. The thermal dependence of biological traits: Ecological ArchivesE094-108. *Ecology*. Wiley; 2013; doi: 10.1890/12-2060.1.
29. Dell AI, Pawar S, Savage VM. Systematic variation in the thermal dependence of physiological and ecological traits. *Proc Natl Acad Sci U S A*. 108:10591–62011;
30. : VectorByte : VecTraits Column definitions. https://vectorbyte.crc.nd.edu/vectraits-columndefs Accessed 2025 Dec 3.
31. Huxley PJ, Murray KA, Pawar S, Cator LJ. The effect of resource limitation on the temperature dependence of mosquito population fitness. *Proc Biol Sci*. The Royal Society; 2021; doi: 10.1098/rspb.2020.3217.
32. VectorByte, dataset 572. https://vectorbyte.crc.nd.edu/vectraits-dataset/572 Accessed 2025 Dec 3.

Figure 1

Click here to access/download;Figure;Fig1.png

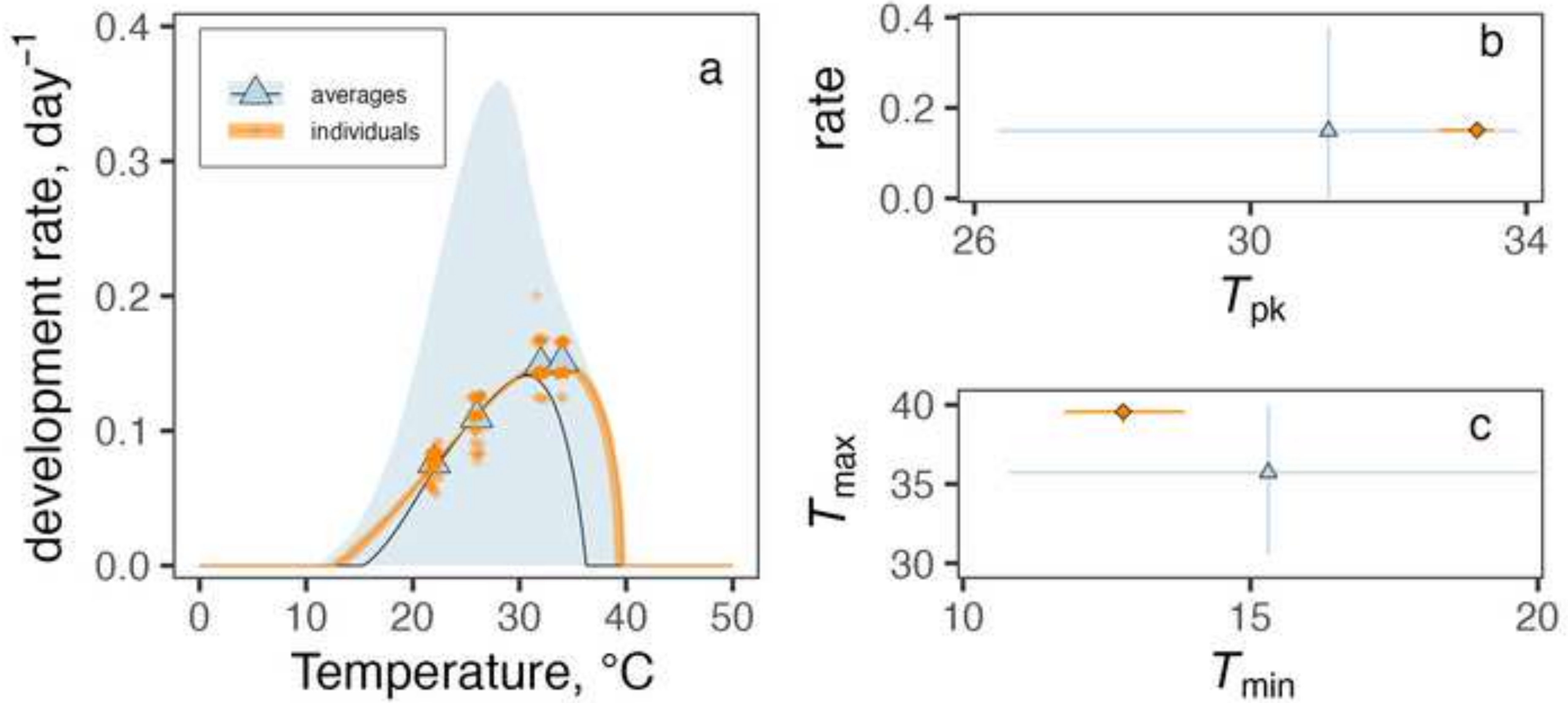

Schematic Figure

Click here to access/download
Supplementary Material
schematicFig.png

Personal Cover Click here to access/download;Personal Cover;MireVTD_Rev1_RESPONSE_CL.pdf

UF UNIVERSITY of FLORIDA
Geography
University of Florida

January 24, 2026

Dear Dr Fan,

Please find enclosed our revised manuscript “MIReVTD, a Minimum Information Standard for Reporting Vector Trait Data” (GIG-D-25-00263), for publication in GigaScience. We appreciate the minor revisions recommended by the reviewers and editor, and detail below our responses (*in italics*) to all their comments.

Editorial:

All web links and URLs should be given a reference number and included in the reference list rather than within the text of the manuscript. Please remove the URLs, cite them as reference and adjust the order of the reference accordingly. Except for “Abstract” and “Availability of Source Code and Requirements"

We have made the requested changes

Reviewer #1:

The authors propose MIReVTD, a concise minimum-information checklist for reporting vector trait data, motivated by the lack of consistent terminology and metadata that impedes reuse and synthesis across studies. The scope and intent are clearly stated in the Abstract and Introduction, including the emphasis on FAIR principles and the illustrative Aedes aegypti example and VecTraits implementation. Overall, this is a timely, valuable contribution that complements MIReAD (arthropod abundance) and the vector competence minimum data standard, and it will be highly useful to both experimentalists and modellers.

We thank the reviewer for this comment

Major

- It would be highly beneficial to demonstrate the compatibility and added value of the MIReVTD and the VecTraits database to the existing initiatives aiming to collect and structure similar information. The authors mentioned ETS, MIAPPE, and MIReAD but and explicit mapping of the minimum information field alignment will help to place MIReVTD in context and facilitate adoption of this standard.

We appreciate this comment, and in our introduction, we have added further description of how this differs from prior minimum standards at lines 98-102 to clarify the intent. An explicit multi-standard mapping is outside the scope of the current effort.

- The "Axes of Variation" section is strong, but it could be clearer about what constitutes a stressor or condition. It would help to list common confounders such as humidity, photoperiod, diet or food ration and quality, larval density, and light cycle, and to encourage recording fixed or background conditions in separate fields rather than only gradients. This would help avoid ambiguity between variables that are experimentally varied and those that simply describe the environment.

We thank the reviewer for these excellent points. We realise that we had included our description of “fixed or background conditions”, which we refer to as ‘additional experimental settings’ - that is, those not being

manipulated - within the description of Axes of Variation. After debating inclusion of another section of explanation outside the original description, we have instead clarified a little further the intent, and the difference (Lines 130-141)

In Figure 2, the second stressor appears to take a fixed value (0.1). This is somewhat confusing because it is not clear whether this field is meant for another gradient (e.g., temperature in the range of 20 to 40 °C in addition to food ration categories), or whether it lists fixed conditions under which the experiment was performed. If it is the latter, it might be more practical to include additional fields for stressors so that all relevant conditions, such as humidity and photoperiod, can be recorded. It would also help to clarify whether a third or further stressor can be added to the table, and how these would appear.

In this specific case, we apologize that this example may create confusion - the stressor that the reviewer refers to is the first treatment level in a series of levels in the experiment. We have updated the figure legend to point out that we only show the first treatment level.

It might in fact be preferable not to distinguish gradients from fixed conditions at all, and instead to treat them uniformly as conditions, each defined with its corresponding unit and uncertainty. This would simplify the structure and prevent confusion about whether a variable was held constant or systematically varied.

As described above, the purpose here is to provide a flexible structure that allows for additional columns. As the reviewer points out, these are all conditions, and we do not a priori distinguish columns as fixed or varying conditions, but leave these specifications to the user/data curator per experiment.

- It would highly improve usability and adoption if the standard also recommended ORCIDs for contributors, DOIs for datasets, and an explicit data license (e.g., CC BY/CC0). If this extension is possible, I recommend that the authors add a short "Data licensing & citation" paragraph to the Results section.

We appreciate this comment and have added this paragraph (lines 291-299) and described the suggested citation/attributions for the data sets in the database. In short, we include the study DOI (at publication) with each row of data, and a column to identify who entered/imported that row. If the data are a group of data as part of a metadata study or aggregation of data, we recommend citing that DOI, to reflect the effort of the aggregating authors. These citation metadata are automatically generated at download from the database for data users to incorporate into their work. The overall dataset (VecTraits) hosted at VectorByte, is CC-BY-NC, and has its own DOI as cited in the paper.

Minor

- Line 248: Fig. 2?

We thank the reviewer for highlighting a typo on our part. In the first sentence of the section titled "Example dataset," "Fig.1" should reference "Fig 2." instead.

- Please update the citation of bayesTPC.

bayesTPC is now published and we have updated the reference.

- If possible, please provide a code snippet with the data used (in Zenodo or as Supplementary Material) for Fig. 1.

Thank you, the code is mentioned in the figure legend and is available at https://vectorbyteorg.github.io/MireVTD-fig-1/

- I believe the followings are also relevant to this study and should be mentioned appropriately:

- Adams B, Franz N, König-Ries B, et al. TraitBank: Practical semantics for organism attribute data. Semantic Web. 2015;7(6):577-588. doi:10.3233/SW-150190

- Kattge, J., Ogle, K., Bönisch, G., Díaz, S., Lavorel, S., Madin, J., Nadrowski, K., Nöllert, S., Sartor, K. and Wirth, C. (2011), A generic structure for plant trait databases. Methods in Ecology and Evolution, 2: 202-213.

Thank you for pointing these out, and we have included them as examples of additional trait database efforts.

Reviewer #2: I read with interest the manuscript as I wholeheartedly agree there is a strong need for harmonization on reporting quantitative measurements of vector traits, especially for the subsequent development of mathematical models.

The paper is well written, and examples are very helpful, particularly the one shown in Figure 1, advocating for the need for the sharing of individual (possibly raw) observations. I have some very minor comments and suggestions.

Thank you for the comments

Given the broad readership of the journal, I feel the Introduction would benefit from some definitions of what the authors mean by vector and vector-borne diseases, with some examples (WNV, DENV, … up to you).

We thank the reviewer for this suggestion and now provide some examples in the Introduction (lines 57-60)

It's not very clear to me how the authors' current proposal aligns with what already proposed in Wu et al. 2022 (ref 21). It seems like some sort of extension? Could you please further elaborate on this?

In response to this and reviewer 1's comment, we have provided clarification in lines 98-102 of the Introduction. We do not want to detract from presenting the standard itself with lengthy comparisons and elaborations, but appreciate the opportunity to clarify.

Regarding latitude and longitude, I think also the coordinate reference system should be standardized (WGS, no UTM or others).

As this is a minimum standard for data entry, we err on the side of ensuring the input datum and resolution is specified, which allows for later users to perform their own spatial standardization and conversion steps. We can encourage a standard system, but this is not the intent of this effort.

You might provide some examples of online repositories (line 187). Some (like GitHub) might not be perpetually available, differently from (hopefully) others like Zenodo or the Supplementary Materials accompanying the paper. The latter might be preferrable in my opinion.

We are not entirely sure to what this comment is referring, but appreciate the thought that online repositories may not be available forever, and we would argue in fact that in many cases Supplementary Material or Appendices can be even shorter lived as journals move between publishers, and in our experience, components can become disconnected in the process, and appendices and supplementary can become irretrievable a decade later. (SJR Personal Experience).

Figure 1. Please provide the equation of the TPC.

We thank the reviewer for this comment. The code for constructing Figure 1 is hosted at https://vectorbyteorg.github.io/MireVTD-fig-1/

Please note that Figure 2 currently does not seem to be cited in the main text (perhaps it should be on line 248?). What does "Dataset: 572" mean?

We thank the reviewer for highlighting a typo on our part. In the first sentence of the section titled "Example dataset," "Fig.1" should reference "Fig 2." instead.

We have clarified in the legend now that Dataset 572 refers to the VectorByte VecTraits data ID number.

As currently VecTraits seem the best (and only?) example of what the authors are proposing, perhaps it should be mentioned in the Abstract as well.

We appreciate this comment, but feel that the paper itself underscores the point, and could not see an easy way to incorporate this into the Abstract.

Lastly, during revision, one author (PH) developed an additional illustrative schematic of the MIReVTD reporting standard, shown here:

Optional schematic

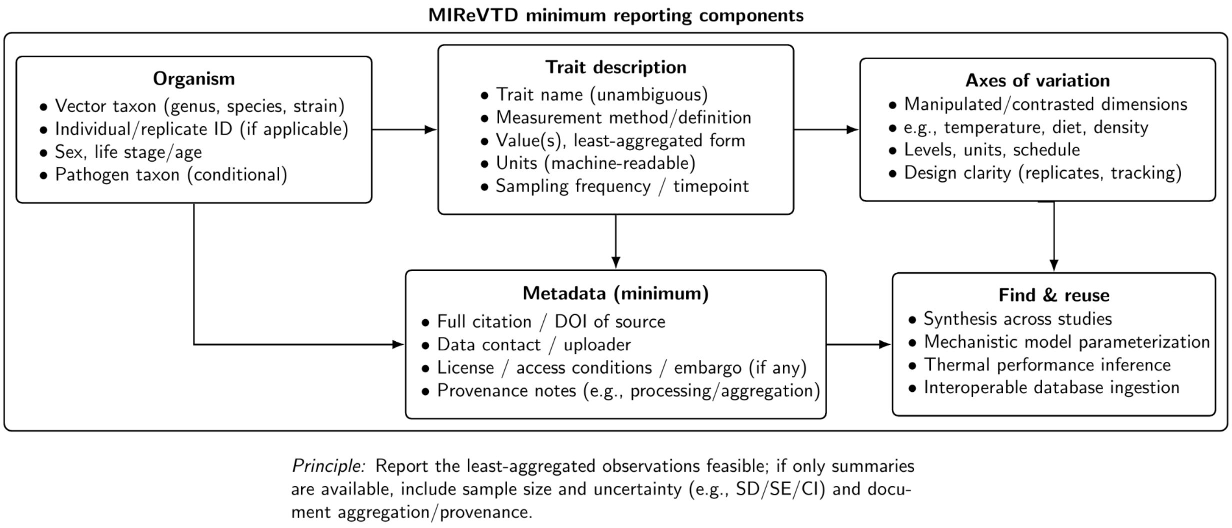

Fig. x. **Schematic overview of the MIReVTD minimum information standard.** Conceptual schematic illustrating the minimum reporting components of MIReVTD (Minimum Information standard for Reporting Vector Trait Data). Vector trait datasets should minimally report (i) Organism information, including vector

taxonomy, life stage, sex, and relevant pathogen identity; (ii) Trait description, specifying the trait measured, how it was measured, the least-aggregated values available, and units; and (iii) Axes of variation, describing experimental or observational dimensions (e.g., temperature, resource level, age) across which the trait was measured. These core components are supported by Metadata, including source citation, provenance, and access information, to enable data discovery and attribution. When reported in this form, vector trait data are findable, interpretable, and reusable for synthesis, database integration, and mechanistic modeling applications.

While we are aware this is redundant with current narrative content and adds length to the submission, if its inclusion in the manuscript is welcome, we would like editorial input on best placement.

We feel that the reviewer feedback facilitated clarification of the manuscript, and hope you now find it suitable for publication in *GigaScience*.

Please do not hesitate to contact me, sjryan@ufl.edu for any further information.
On behalf of the authors,

Sincerely,

Sadie Ryan, PhD
Professor, Medical Geography
