# Supplementary material for: MIReVTD, a minimum information standard for reporting vector trait data
Source: Gigascience. 2026 Feb 28;15:giag020. doi: 10.1093/gigascience/giag020 (PMC13122843; doi:10.1093/gigascience/giag020)
Supplement: giag020_Authors_Response_To_Reviewer_Comments_original_submission [file giag020_authors_response_to_reviewer_comments_original_submission.pdf]

Author's Response To Reviewer Comments

Full response included in Personal Cover letter as well - content pasted here from letter. Thanks.

January 24, 2026

Dear Dr Fan,
Please find enclosed our revised manuscript “MIReVTD, a Minimum Information Standard for Reporting Vector Trait Data” (GIG-D-25-00263), for publication in GigaScience. We appreciate the minor revisions recommended by the reviewers and editor, and detail below our responses (in italics) to all their comments.
Editorial:
All web links and URLs should be given a reference number and included in the reference list rather than within the text of the manuscript. Please remove the URLs, cite them as reference and adjust the order of the reference accordingly. Except for “Abstract” and “Availability of Source Code and Requirements"
We have made the requested changes
Reviewer #1:
The authors propose MIReVTD, a concise minimum-information checklist for reporting vector trait data, motivated by the lack of consistent terminology and metadata that impedes reuse and synthesis across studies. The scope and intent are clearly stated in the Abstract and Introduction, including the emphasis on FAIR principles and the illustrative Aedes aegypti example and VecTraits implementation. Overall, this is a timely, valuable contribution that complements MIReAD (arthropod abundance) and the vector competence minimum data standard, and it will be highly useful to both experimentalists and modellers.
We thank the reviewer for this comment
Major
- It would be highly beneficial to demonstrate the compatibility and added value of the MIReVTD and the VecTraits database to the existing initiatives aiming to collect and structure similar information. The authors mentioned ETS, MIAPPE, and MIReAD but and explicit mapping of the minimum information field alignment will help to place MIReVTD in context and facilitate adoption of this standard.
We appreciate this comment, and in our introduction, we have added further description of how this differs from prior minimum standards at lines 98-102 to clarify the intent. An explicit multi-standard mapping is outside the scope of the current effort.
- The "Axes of Variation" section is strong, but it could be clearer about what constitutes a stressor or condition. It would help to list common confounders such as humidity, photoperiod, diet or food ration and quality, larval density, and light cycle, and to encourage recording fixed or background conditions in separate fields rather than only gradients. This would help avoid ambiguity between variables that are experimentally varied and those that simply describe the environment.
We thank the reviewer for these excellent points. We realise that we had included our description of “fixed or background conditions”, which we refer to as ‘additional experimental settings’ - that is, those not being manipulated - within the description of Axes of Variation. After debating inclusion of another section of explanation outside the original description, we have instead clarified a little further the intent, and the difference (Lines 130-141)
In Figure 2, the second stressor appears to take a fixed value (0.1). This is somewhat confusing because it is not clear whether this field is meant for another gradient (e.g., temperature in the range of 20 to 40 °C in addition to food ration categories), or whether it lists fixed conditions under which the experiment was performed. If it is the latter, it might be more practical to include additional fields for stressors so that all relevant conditions, such as humidity and photoperiod, can be recorded. It would also help to clarify whether a third or further stressor can be added to the table, and how these would appear.
In this specific case, we apologize that this example may create confusion - the stressor that the reviewer refers to is the first treatment level in a series of levels in the experiment. We have updated the figure legend to point out that we only show the first treatment level.
It might in fact be preferable not to distinguish gradients from fixed conditions at all, and instead to treat them uniformly as conditions, each defined with its corresponding unit and uncertainty. This would simplify the structure and prevent confusion about whether a variable was held constant or systematically varied.

As described above, the purpose here is to provide a flexible structure that allows for additional columns. As the reviewer points out, these are all conditions, and we do not a priori distinguish columns as fixed or varying conditions, but leave these specifications to the user/data curator per experiment.
- It would highly improve usability and adoption if the standard also recommended ORCIDs for contributors, DOIs for datasets, and an explicit data license (e.g., CC BY/CC0). If this extension is possible, I recommend that the authors add a short "Data licensing & citation" paragraph to the Results section.
We appreciate this comment and have added this paragraph (lines 291-299) and described the suggested citation/attributions for the data sets in the database. In short, we include the study DOI (at publication) with each row of data, and a column to identify who entered/imported that row. If the data are a group of data as part of a metadata study or aggregation of data, we recommend citing that DOI, to reflect the effort of the aggregating authors. These citation metadata are automatically generated at download from the database for data users to incorporate into their work. The overall dataset (VecTraits) hosted at VectorByte, is CC-BY-NC, and has its own DOI as cited in the paper.
Minor
- Line 248: Fig. 2?
We thank the reviewer for highlighting a typo on our part. In the first sentence of the section titled “Example dataset,” “Fig.1” should reference “Fig 2.” instead.
- Please update the citation of bayesTPC.
bayesTPC is now published and we have updated the reference.
- If possible, please provide a code snippet with the data used (in Zenodo or as Supplementary Material) for Fig. 1.
Thank you, the code is mentioned in the figure legend and is available at https://vectorbyteorg.github.io/MireVTD-fig-1/
- I believe the followings are also relevant to this study and should be mentioned appropriately:
- Adams B, Franz N, König-Ries B, et al. TraitBank: Practical semantics for organism attribute data. Semantic Web. 2015;7(6):577-588. doi:10.3233/SW-150190
- Kattge, J., Ogle, K., Bönisch, G., Díaz, S., Lavorel, S., Madin, J., Nadrowski, K., Nöllert, S., Sartor, K. and Wirth, C. (2011), A generic structure for plant trait databases. Methods in Ecology and Evolution, 2: 202-213.
Thank you for pointing these out, and we have included them as examples of additional trait database efforts.
Reviewer #2: I read with interest the manuscript as I wholeheartedly agree there is a strong need for harmonization on reporting quantitative measurements of vector traits, especially for the subsequent development of mathematical models.
The paper is well written, and examples are very helpful, particularly the one shown in Figure 1, advocating for the need for the sharing of individual (possibly raw) observations. I have some very minor comments and suggestions.
Thank you for the comments
Given the broad readership of the journal, I feel the Introduction would benefit from some definitions of what the authors mean by vector and vector-borne diseases, with some examples (WNV, DENV, … up to you).
We thank the reviewer for this suggestion and now provide some examples in the Introduction (lines 57-60)
It's not very clear to me how the authors' current proposal aligns with what already proposed in Wu et al. 2022 (ref 21). It seems like some sort of extension? Could you please further elaborate on this?
In response to this and reviewer 1’s comment, we have provided clarification in lines 98-102 of the Introduction. We do not want to detract from presenting the standard itself with lengthy comparisons and elaborations, but appreciate the opportunity to clarify.
Regarding latitude and longitude, I think also the coordinate reference system should be standardized (WGS, no UTM or others).
As this is a minimum standard for data entry, we err on the side of ensuring the input datum and resolution is specified, which allows for later users to perform their own spatial standardization and conversion steps. We can encourage a standard system, but this is not the intent of this effort.
You might provide some examples of online repositories (line 187). Some (like GitHub) might not be perpetually available, differently from (hopefully) others like Zenodo or the Supplementary Materials accompanying the paper. The latter might be preferrable in my opinion.
We are not entirely sure to what this comment is referring, but appreciate the thought that online

repositories may not be available forever, and we would argue in fact that in many cases Supplementary Material or Appendices can be even shorter lived as journals move between publishers, and in our experience, components can become disconnected in the process, and appendices and supplementary can become irretrievable a decade later. (SJR Personal Experience).
Figure 1. Please provide the equation of the TPC.
We thank the reviewer for this comment. The code for constructing Figure 1 is hosted at https://vectorbyteorg.github.io/MireVTD-fig-1/
Please note that Figure 2 currently does not seem to be cited in the main text (perhaps it should be on line 248?). What does "Dataset: 572" mean?
We thank the reviewer for highlighting a typo on our part. In the first sentence of the section titled "Example dataset," "Fig.1" should reference "Fig 2." instead.
We have clarified in the legend now that Dataset 572 refers to the VectorByte VecTraits data ID number.
As currently VecTraits seem the best (and only?) example of what the authors are proposing, perhaps it should be mentioned in the Abstract as well.
We appreciate this comment, but feel that the paper itself underscores the point, and could not see an easy way to incorporate this into the Abstract.
Lastly, during revision, one author (PH) developed an additional illustrative schematic of the MIReVTD reporting standard, shown here:

Optional schematic

Fig. x. Schematic overview of the MIReVTD minimum information standard. Conceptual schematic illustrating the minimum reporting components of MIReVTD (Minimum Information standard for Reporting Vector Trait Data). Vector trait datasets should minimally report (i) Organism information, including vector taxonomy, life stage, sex, and relevant pathogen identity; (ii) Trait description, specifying the trait measured, how it was measured, the least-aggregated values available, and units; and (iii) Axes of variation, describing experimental or observational dimensions (e.g., temperature, resource level, age) across which the trait was measured. These core components are supported by Metadata, including source citation, provenance, and access information, to enable data discovery and attribution. When reported in this form, vector trait data are findable, interpretable, and reusable for synthesis, database integration, and mechanistic modeling applications.
While we are aware this is redundant with current narrative content and adds length to the submission, if its inclusion in the manuscript is welcome, we would like editorial input on best placement.
We feel that the reviewer feedback facilitated clarification of the manuscript, and hope you now find it suitable for publication in GigaScience.

Please do not hesitate to contact me, sjryan@ufl.edu for any further information.
On behalf of the authors,

Sincerely,

Sadie Ryan, PhD
Professor, Medical Geography

Cookie Preference Center

We use cookies which are necessary to make our site work. We may also use additional cookies to analyze, improve and personalize our content and your digital experience. For more information, see our Cookie Policy.

You may choose not to allow some types of cookies. However, blocking some types may impact your experience of our site and the services we are able to offer. See the different category headings below to find out more or change your settings.

Allow all

Manage Consent Preferences

Strictly Necessary Cookies

Always active

These cookies are necessary for the website to function and cannot be switched off in our systems. They are usually only set in response to actions made by you which amount to a request for services, such as setting your privacy preferences, logging in or filling in forms. You can set your browser to block or alert you about these cookies, but some parts of the site will not then work.

Functional Cookies

These cookies enable the website to provide enhanced functionality and personalisation. They may be set by us or by third party providers whose services we have added to our pages. If you do not allow these cookies then some or all of these services may not function properly.

Performance Cookies

These cookies allow us to count visits and traffic sources so we can measure and improve the performance of our site. They help us to know which pages are the most and least popular and see how visitors move around the site.

Targeting Cookies

These cookies may be set through our site by our advertising partners. They may be used by those companies to build a profile of your interests and show you relevant adverts on other sites. If you do not allow these cookies, you will experience less targeted advertising.

Cookie List

Clear

Apply Cancel
Consent Leg.Interest

Confirm my choices

Power
