# Supplementary material for: MIReVTD, a minimum information standard for reporting vector trait data
Source: Gigascience. 2026 Feb 28;15:giag020. doi: 10.1093/gigascience/giag020 (PMC13122843; doi:10.1093/gigascience/giag020)
Supplement: giag020_Reviewer_1_Report_original_submission — Reviewer 1 -- 10/6/2025 [file giag020_reviewer_1_report_original_submission.pdf]

Reviewer Report

Title: MIReVTD, a Minimum Information Standard for Reporting Vector Trait Data

Version: Original Submission **Date:** 10/6/2025

Reviewer name: Reviewer 1

Reviewer Comments to Author:

The authors propose MIReVTD, a concise minimum-information checklist for reporting vector trait data, motivated by the lack of consistent terminology and metadata that impedes reuse and synthesis across studies. The scope and intent are clearly stated in the Abstract and Introduction, including the emphasis on FAIR principles and the illustrative Aedes aegypti example and VecTraits implementation. Overall, this is a timely, valuable contribution that complements MIReAD (arthropod abundance) and the vector competence minimum data standard, and it will be highly useful to both experimentalists and modellers.Major- It would be highly beneficial to demonstrate the compatibility and added value of the MIReVTD and the VecTraits database to the existing initiatives aiming to collect and structure similar information. The authors mentioned ETS, MIAPPE, and MIReAD but and explicit mapping of the minimum information field alignment will help to place MIReVTD in context and facilitate adoption of this standard.- The "Axes of Variation" section is strong, but it could be clearer about what constitutes a stressor or condition. It would help to list common confounders such as humidity, photoperiod, diet or food ration and quality, larval density, and light cycle, and to encourage recording fixed or background conditions in separate fields rather than only gradients. This would help avoid ambiguity between variables that are experimentally varied and those that simply describe the environment.â€¨In Figure 2, the second stressor appears to take a fixed value (0.1). This is somewhat confusing because it is not clear whether this field is meant for another gradient (e.g., temperature in the range of 20 to 40 Â°C in addition to food ration categories), or whether it lists fixed conditions under which the experiment was performed. If it is the latter, it might be more practical to include additional fields for stressors so that all relevant conditions, such as humidity and photoperiod, can be recorded. It would also help to clarify whether a third or further stressor can be added to the table, and how these would appear.â€¨It might in fact be preferable not to distinguish gradients from fixed conditions at all, and instead to treat them uniformly as conditions, each defined with its corresponding unit and uncertainty. This would simplify the structure and prevent confusion about whether a variable was held constant or systematically varied.- It would highly improve usability and adoption if the standard also recommended ORCIDs for contributors, DOIs for datasets, and an explicit data license (e.g., CC BY/CC0). If this extension is possible, I recommend that the authors add a short "Data licensing & citation" paragraph to the Results section.Minor- Line 248: Fig. 2?- Please update the citation of bayesTPC.- If possible, please provide a code snippet with the data used (in Zenodo or as Supplementary Material) for Fig. 1.- I believe the followings are also relevant to this study and should be mentioned appropriately:- Adams B, Franz N, KÃ¶nig-Ries B, et al. TraitBank: Practical semantics for organism attribute data. Semantic Web. 2015;7(6):577-588. doi:10.3233/SW-150190- Kattge, J., Ogle, K., BÃ¶nisch, G., DÃaz, S., Lavorel, S., Madin, J., Nadrowski, K., NÃ¶llert, S., Sartor, K. and Wirth, C. (2011), A generic structure for plant trait

databases. Methods in Ecology and Evolution, 2: 202-213. https://doi.org/10.1111/j.2041-210X.2010.00067.x

Level of Interest

Please indicate how interesting you found the manuscript: Choose an item.

Quality of Written English

Please indicate the quality of language in the manuscript: Choose an item.

Declaration of Competing Interests

Please complete a declaration of competing interests, considering the following questions:

- Have you in the past five years received reimbursements, fees, funding, or salary from an organisation that may in any way gain or lose financially from the publication of this manuscript, either now or in the future?
- Do you hold any stocks or shares in an organisation that may in any way gain or lose financially from the publication of this manuscript, either now or in the future?
- Do you hold or are you currently applying for any patents relating to the content of the manuscript?
- Have you received reimbursements, fees, funding, or salary from an organization that holds or has applied for patents relating to the content of the manuscript?
- Do you have any other financial competing interests?
- Do you have any non-financial competing interests in relation to this paper?

If you can answer no to all of the above, write 'I declare that I have no competing interests' below. If your reply is yes to any, please give details below.

I declare that I have no competing interests

I agree to the open peer review policy of the journal. I understand that my name will be included on my report to the authors and, if the manuscript is accepted for publication, my named report including any attachments I upload will be posted on the website along with the authors' responses. I agree for my report to be made available under an Open Access Creative Commons CC-BY license (http://creativecommons.org/licenses/by/4.0/). I understand that any comments which I do not wish to be included in my named report can be included as confidential comments to the editors, which will not be published.

Choose an item.

To further support our reviewers, we have joined with Publons, where you can gain additional credit to further highlight your hard work (see: https://publons.com/journal/530/gigascience). On publication of this paper, your review will be automatically added to Publons, you can then choose whether or not to claim your Publons credit. I understand this statement.

Yes Choose an item.
