# Supplementary material for: MIReVTD, a minimum information standard for reporting vector trait data
Source: Gigascience. 2026 Feb 28;15:giag020. doi: 10.1093/gigascience/giag020 (PMC13122843; doi:10.1093/gigascience/giag020)
Supplement: giag020_Reviewer_2_Report_original_submission — Reviewer 2 -- 11/5/2025 [file giag020_reviewer_2_report_original_submission.pdf]

Reviewer Report

Title: MIReVTD, a Minimum Information Standard for Reporting Vector Trait Data

Version: Original Submission **Date:** 11/5/2025

Reviewer name: Reviewer 2

Reviewer Comments to Author:

I read with interest the manuscript as I wholeheartedly agree there is a strong need for harmonization on reporting quantitative measurements of vector traits, especially for the subsequent development of mathematical models.The paper is well written, and examples are very helpful, particularly the one shown in Figure 1, advocating for the need for the sharing of individual (possibly raw) observations. I have some very minor comments and suggestions.Given the broad readership of the journal, I feel the Introduction would benefit from some definitions of what the authors mean by vector and vector-borne diseases, with some examples (WNV, DENV, … up to you).It's not very clear to me how the authors' current proposal aligns with what already proposed in Wu et al. 2022 (ref 21). It seems like some sort of extension? Could you please further elaborate on this?Regarding latitude and longitude, I think also the coordinate reference system should be standardized (WGS, no UTM or others).You might provide some examples of online repositories (line 187). Some (like GitHub) might not be perpetually available, differently from (hopefully) others like Zenodo or the Supplementary Materials accompanying the paper. The latter might be preferrable in my opinion.Figure 1. Please provide the equation of the TPC.Please note that Figure 2 currently does not seem to be cited in the main text (perhaps it should be on line 248?). What does "Dataset: 572" mean?As currently VecTraits seem the best (and only?) example of what the authors are proposing, perhaps it should be mentioned in the Abstract as well.

Level of Interest

Please indicate how interesting you found the manuscript: Choose an item.

Quality of Written English

Please indicate the quality of language in the manuscript: Choose an item.

Declaration of Competing Interests

Please complete a declaration of competing interests, considering the following questions:

- Have you in the past five years received reimbursements, fees, funding, or salary from an organisation that may in any way gain or lose financially from the publication of this manuscript, either now or in the future?
- Do you hold any stocks or shares in an organisation that may in any way gain or lose financially from the publication of this manuscript, either now or in the future?

- Do you hold or are you currently applying for any patents relating to the content of the manuscript?
- Have you received reimbursements, fees, funding, or salary from an organization that holds or has applied for patents relating to the content of the manuscript?
- Do you have any other financial competing interests?
- Do you have any non-financial competing interests in relation to this paper?

If you can answer no to all of the above, write 'I declare that I have no competing interests' below. If your reply is yes to any, please give details below.

I declare that I have no competing interests

I agree to the open peer review policy of the journal. I understand that my name will be included on my report to the authors and, if the manuscript is accepted for publication, my named report including any attachments I upload will be posted on the website along with the authors' responses. I agree for my report to be made available under an Open Access Creative Commons CC-BY license (http://creativecommons.org/licenses/by/4.0/). I understand that any comments which I do not wish to be included in my named report can be included as confidential comments to the editors, which will not be published.

Choose an item.

To further support our reviewers, we have joined with Publons, where you can gain additional credit to further highlight your hard work (see: https://publons.com/journal/530/gigascience). On publication of this paper, your review will be automatically added to Publons, you can then choose whether or not to claim your Publons credit. I understand this statement.

Yes Choose an item.
